# Using an Artificial Neural Network to Predict Coronary Microvascular Obstruction (No-Reflow Phenomenon) during Percutaneous Coronary Interventions in Patients with Myocardial Infarction

**DOI:** 10.17691/stm2021.13.6.01

**Published:** 2021-12-28

**Authors:** A.A. Frolov, I.G. Pochinka, B.E. Shakhov, A.S. Mukhin, I.A. Frolov, M.K. Barinova, E.G. Sharabrin

**Affiliations:** Assistant, Department of Hospital Surgery named after B.A. Korolev; Privolzhsky Research Medical University, 10/1 Minin and Pozharsky Square, Nizhny Novgorod, 603005, Russia; Physician, Department of Radiosurgery Methods in Diagnostics and Therapy; City Clinical Hospital No.13, 51 Patriotov St., Nizhny Novgorod, 603018, Russia;; Associate Professor, Department of Endocrinology and Internal Diseases; Privolzhsky Research Medical University, 10/1 Minin and Pozharsky Square, Nizhny Novgorod, 603005, Russia; Head of the Cardiology Department; City Clinical Hospital No.13, 51 Patriotov St., Nizhny Novgorod, 603018, Russia;; Professor, Head of the Department of X-ray Endovascular Diagnosis and Treatment; Privolzhsky Research Medical University, 10/1 Minin and Pozharsky Square, Nizhny Novgorod, 603005, Russia;; Professor, Head of Department of Hospital Surgery named after B.A. Korolev; Privolzhsky Research Medical University, 10/1 Minin and Pozharsky Square, Nizhny Novgorod, 603005, Russia;; Physician, Department of Radiosurgery Methods in Diagnostics and Therapy; City Clinical Hospital No.13, 51 Patriotov St., Nizhny Novgorod, 603018, Russia;; Researcher, International Laboratory of Dynamic Systems and Applications; Nizhny Novgorod Branch of National Research University “Higher School of Economics”, 30 Sormovskoe shosse, Nizhny Novgorod, 603014, Russia; Professor, Department of X-ray Endovascular Diagnosis and Treatment; Privolzhsky Research Medical University, 10/1 Minin and Pozharsky Square, Nizhny Novgorod, 603005, Russia;

**Keywords:** myocardial infarction, coronary microvascular obstruction, no-reflow, percutaneous coronary intervention, artificial neural network, logistic regression, machine learning

## Abstract

**The aim of the study** was to develop, evaluate, and validate an artificial neural network to predict coronary microvascular obstruction (CMVO) during percutaneous coronary interventions (PCI) in patients with myocardial infarctions (MI) based on the parameters, which are routinely available in an operating room when choosing a surgical approach.

**Materials and Methods:**

5621 patients with MI and emergency PCI were retrospectively selected from the database of the City Clinical Hospital No.13 (Nizhny Novgorod, Russia); among them, there were 3935 men (70%) and 1686 women (30%), their mean age was 61.5±10.8 years. CMVO was recorded in 201 (4%) patients (the blood flow in the infarction-related artery after PCI was less than 3 points according to TIMI flow grade). The following input parameters were assessed: age, gender, past history of coronary artery disease, previous revascularization, presence of ST-segment elevation, a class of acute heart failure, a fact of systemic thrombolytic therapy administration and its effectiveness, symptom-to-balloon time, severity of coronary thrombosis and atherosclerosis, the number of stents and the number of operated coronary arteries. The sampling was divided into a training group (n=4060), a testing group (n=717), and an independent validation group (n=844).

**Results:**

We developed an artificial neural network by a fully connected multilayer perception with forward signal propagation and two hidden layers (the area under the ROC curve — 0.69) to predict CMVO based on the subsampling for training and testing. The network model was tested on an independent subsampling (the area under the ROC curve — 0.64, negative predictive value — 97.4%, positive predictive value — 14.6%).

**Conclusion:**

The developed artificial neural network enables to use the parameters routinely available in an operating room when choosing a surgical approach and predict CMVO development during PCI in MI patients with accuracy sufficient for practical use.

## Introduction

By coronary microvascular obstruction (CMVO, a no-reflow phenomenon) we mean an inadequate myocardial perfusion after successful recanalization of the coronary artery (CA) operated during percutaneous coronary intervention (PCI). The phenomenon is still one of the most common complications in endovascular treatment of myocardial infarction (MI) and occurs in about 10% patients in case of using angiographic criteria [1]. Formidable difficulties in CMVO prognosis and therapy, to a greater degree, are due to the fact that several mechanisms underlie its pathogenesis; and the contribution of the mechanisms to its development varies considerably in different patients [2].

Currently, the most promising technique to predict the phenomenon is a prognostic model by Wang et al. [3]; the method was developed by a logistic regression and is characterized by rather high quality (the area under ROC curve is 0.800; confidential interval is 95% (95% CI) — 0.772–0.826), and acceptable sensitivity and specificity values (76.1 and 70.8%, respectively). However, the model has a number of restrictions: the use of laboratory markers, which are usually unavailable in case of emergency MI patients, and no validation using independent data.

One of the relevant approaches that can provide a prognostic model compatible in efficiency and lacking the above-mentioned limitations is the analysis of a large bulk of data using machine learning techniques, in particular — artificial neural networks (ANN).

**The aim of the study** was to develop, evaluate, and validate an artificial neural network to predict coronary microvascular obstruction (no-reflow phenomenon) during percutaneous coronary interventions in patients with myocardial infarctions based on the parameters, which are routinely available in an operating room when choosing a surgical approach.

## Materials and Methods

### Sampling description

To create ANN, we retrospectively studied the data of 19,596 patients from the register of treatment and hospital outcomes of patients with acute coronary syndrome in the City Clinical Hospital No.13 (Nizhny Novgorod, Russia) over the period of 2011–2020. Before selection and statistical processing, the information about patients was anonymized.

**Figure 1.**
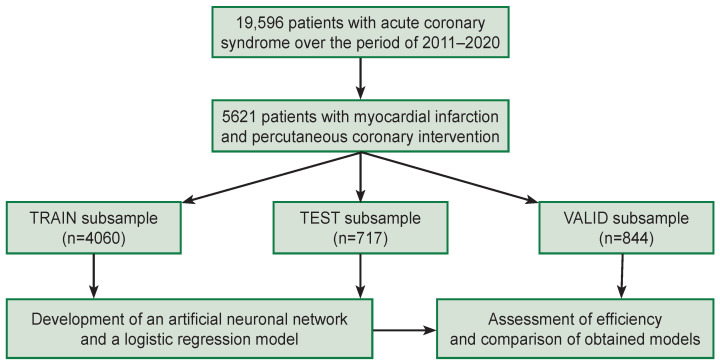
Scheme of patients’ inclusion into the study and development of prognostic models Advanced Researches

Inclusion criteria were: confirmed MI and performed PCI. Patients’ files with incomplete data and the files containing the information that should be considered statistically as “an outlying case” were excluded from the subsequent analysis. Finally, 5621 patients were chosen. Figure 1 represents a general diagram of patients’ inclusion into the study and the development of prognostic models.

MI diagnosis was made based on clinical and biochemical criteria according to the third, and then the fourth universal MI definition [4]. PCI consisted in implanting a stent into the infarction-responsible artery (IRA) with residual stenosis of less than 50%. Congestive heart failure (CHF) severity was evaluated according to Killip classification [1, 3], blood flow degree in IRA after PCI — according to thrombolysis in myocardial infarction (TIMI) flow grade [5], IRA thrombosis intensity (after the passage of coronary guide) — according to TIMI thrombus grade [6].

CMVO phenomenon was revealed in 201 patients (4%) from all the subjects involved in the study (n=5621). CMVO development was diagnosed based on blood flow assessment in IRA according to TIMI flow grade as of the moment of PCI completion. CMVO diagnostic criterion was blood flow assessed less than 3 points providing there were no other causes, which could make it decrease (persisting spasm, CA dissection, a large thrombo-embolus, marked residual stenosis).

To create ANN, the following parameters were used: age, gender, angina pectoris and/or past history MI, previous PCI and/or coronary bypass surgery, the presence of steady ST-segment elevation on ECG, CHF class, the fact of performed systemic thrombolytic therapy and its efficiency, symptom-to-balloon time (from anginose status to coronary blood flow recovery during PCI), marked IRA thrombosis (4, 5 degree according to TIMI thrombus grade), severe atherosclerotic CA (one-, two-, three-vessel disease and/or left CA trunk disease), the number of implanted stents and the number of operated CA. Table 1 shows the characteristics of patients’ groups with developed or undeveloped CMVO.

**Table 1:** Characteristics of patient groups with or without coronary microvascular obstruction

Parameter	Patients with developed CMVO (n=201)	Patients with no CMVO (n=5420)	p
Age (years), Me [Q1; Q3]	64.3 [56.5; 72.4]	61.7 [54.7; 68.4]	**0.001**
Males/females, n (%)	140 (69.7)/61 (30.3)	3795 (70.0)/1625 (30.0)	0.911
Past angina pectoris, n (%)	64 (31.8)	1494 (27.6)	0.184
Past myocardial infarction, n (%)	37 (18.4)	779 (14.4)	0.110
Past percutaneous coronary intervention, n (%)	20 (10.0)	392 (7.2)	0.147
Past coronary bypass, n (%)	1 (0.5)	49 (0.9)	0.547
Diabetes mellitus, n (%)	49 (24.4)	1144 (21.1)	0.265
Mortality during hospitalization, n (%)	36 (17.9)	177 (3.3)	**<0.001**
Myocardial infarction with ST-segment elevation, n (%)	181 (90.0)	4167 (76.9)	**<0.001**
CHF, class 4 according to Killip classification, n (%)	28 (13.9)	149 (2.7)	**<0.001**
Effective pre-hospital systemic thrombolytic therapy, n (%)	18 (9.0)	650 (12.0)	0.191
Noneffective pre-hospital systemic thrombolytic therapy, n (%)	49 (24.4)	888 (16.4)	**0.003**
Symptom-to-balloon time (h), Me [Q1; Q3]	9.3 [4.2; 18.0]	9.7 [4.3; 19.5]	**0.041**
TIMI thrombus grade in infarction-responsible artery, IV–V degree, n (%)	43 (21.4)	421 (7.8)	**<0.001**
Three-vessel CA impairment and/or left CA trunk impairment, n (%)	95 (47.3)	2176 (40.1)	**0.043**
PCI using three or more stents, n (%)	31 (15.4)	373 (6.9)	**<0.001**
Single-step PCI on several CA, n (%)	17 (8.5)	416 (7.7)	0.683

Note: CMVO — coronary microvascular obstruction; CHF — congestive heart failure; CA — coronary artery; PCI — percutaneous coronary intervention.

### Statistical processing

To determine the distribution character, we used Kolmogorov–Smirnov test, Mann– Whitney test was used to assess statistical significance of the qualitative data; and to evaluate the significance of qualitative data differences. We used χ^2^ Pearson criterion. The differences were considered statistically significant if p≤0.05. Quantitative data were represented in the form of median and interquartile intervals (Me [Q1; Q3]). The data were statistically processed using Statistica 10.0 (StatSoft Inc., USA) and MedCalc 11.5 (MedCalc Software Ltd, Belgium).

### Data preparation

The patients involved in the study (n=5621) were randomly divided into three subsamples (subgroups): a training subgroup (TRAIN), a control subgroup (TEST), and a valid subgroup (VALID) in the ratio 4060 (72.2%), 717 (12.8%), and 844 (15.0%) patients, respectively (see Figure 1). The number of CMVO patients in TRAIN subsample was 143 (3.5% from 4060 patients), in TEST subsample there were 25 (3.5% from 717) patients, in VALID subsample: 33 (3.9% from 844) patients.

Continuous numeric parameters (age and symptom-to-balloon time) were normalized to a mean value and standard deviation (M±SD) [7]. Mean and standard deviation were calculated based on TRAIN subsample data. The obtained data were used then to normalize the indices in other subsamples. Mean age in TRAIN subgroup was 61.4±10.0 years, mean symptom-to-balloon time was 14.9±14.2 h. Categorical data were re-encoded using a unitary code (one-hot encoding) [7]. In the end, the data were mixed.

Taking into account the imbalance of the data set on CMVO patients’ percentage (only 3.5% in TRAIN subgroup) we applied a number of methods to correct the imbalance of classes in a training subsample. The following techniques were used: random undersampling up to ratio 143/143, random oversampling up to ratio 3917/3917, synthetic minority oversampling technique (SMOTE) up to ratio 3917/3917, training considering correction weighs for predictable classes (0.52 — for patients without CMVO, and 14.20 — for patients with CMVO) [8].

### Structure and training of artificial neural networks

ANN was developed in Python programming environment using machine learning libraries with an open-source code TensorFlow, Keras, and scikit-learn [9]. In order to correct imbalanced initial data, we used the algorithms from imbalanced-learn library [10]. For model training, we used TRAIN subsample, for “convergence” control — TEST subsample. VALID subsample was used subsequently for independent validation (see Figure 1). While developing and training ANN, we probed various versions of network structure and size, activation function and loss function, training and regularization algorithms, as well as various approaches to imbalanced data work.

An optimal ANN has the structure of a fully connected multilayered perceptron (MLP) [11] with direct signaling, the number of inputs — 35, two buried layers (36 and 72 hidden neurons), and the number of inputs — 1 (MLP formula 35-36-72-1). On the first hidden layer, we used a linear function as an activation function, and on the second layer — a function of rectified linear unit (ReLU), on the output element — sigmoid function.

Signal distribution function was standard for the above-mentioned ANN type. For each node of the current layer, the overlying layer node value was multiplied by its weight and was added to the values of other nodes and displacement weight; after that, it was processed by an activation function [11]:


$$f(x_{1}w_{1}+x_{2}w_{2}+...+x_{n}w_{n}+b),$$

where: ƒ — activation function of a current layer; *x* — node value of an overlying layer; *w* — node weight of an overlying layer relating to a current layer; *n* — a number of nodes in an overlying layer; *b* — displacement weight for a current layer.

Obtained ANN made adverse outcome prognosis (CMVO) in the range from 0 to 1. For result classification, the threshold value was used; its elevation was considered that ANN predicted an adverse outcome.

**Figure 2.**
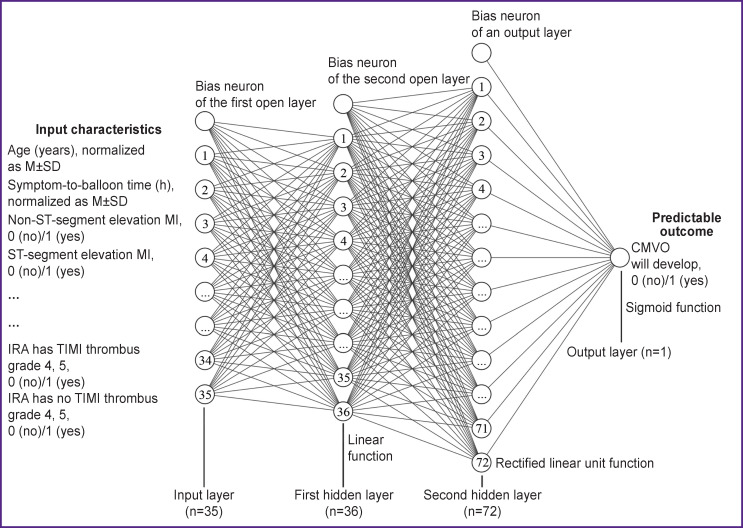
Structural graph of a developed artificial neuronal network n— number of neurons in a layer

Since further network complicacy and size gain did not result in its efficiency improvement, we decided upon the above-described architecture. Figure 2 represents a structure graph of the developed ANN.

To optimize weights during ANN training, we used an algorithm of adaptive moment estimation (Adam) with the rate of learning 0.001 and other parameters by default [12]. As a loss assessment function, we used binary cross-entropy [13]. Within the training, for initial weight initialization, Xavier uniform initialization (Glorot uniform initialization) was applied, and an initial bias was calculated to speed up training [14].

To assess ANN efficiency and search for an optimal cut-off value, we used ROC analysis to evaluate the area under ROC curve (AUC). Based on the obtained cut-off value, an error matrix was constructed to assess metrics. An error matrix included four cells with incidence referred to one of the categories: true positive (TP), false positive (FP), true negative (TN), and false negative (FN). As metrics, we studied sensitivity/recall, specificity, positive predictive value, negative predictive value/precision, *F*_1_-score. The metrics were calculated according to the following formulas:


$$\begin{array}{l} \text{sensitivity = TP/(TP + FN);}\\ \text{specificity = TN/(TN + FP);}\\ \text{positive predictive value = TP/(TP + FP);}\\ \text{negative predictive value/precision = TN/(TN+FN);}\\ F_{\text{1}}\text{-score = 2}\cdot \text{precision}\cdot \text{recall/(precision + recall).} \end{array}$$

In order to prevent ANN retraining, we used intermediate dropout layers with exclusion probability of 0.5, applied L1-regularization of an output layer with a regularization factor of 0.1, and realized an early shutdown function based on *F*_1_-score [15].

The most effective ANN (with maximum *F*_1_-score and AUC) was obtained when training using correction weights for predictable classes. The network was chosen for further work and validation.

### Development of a comparative logistic regression model

For comparative analysis of the obtained ANN efficiency, a logistic regression model was developed:


$$\begin{array}{l} \text{logit (p) = }-\text{6.2683 + 0.03052}\cdot \text{age (years) +}\\ \text{+ 0.9561}\cdot \text{TIMI thrombus grade 4, 5 (yes/no) +}\\ \text{+ 0.6897}\cdot \text{MI with ST-segment elevation (yes/no) +}\\ \text{+ 1.4631}\cdot \text{4 CHF class according to Killip (yes/no) +}\\ \text{+ 0.9496}\cdot \text{implantation of 3 and more stents (yes/no) +}\\ \text{+ 0.5813}\cdot \text{ineffective systemic thrombolysis (yes/no).} \end{array}$$

Here: logit (p) — response variable of logistic regression.

To develop a model, we used a stepwise technique excluding if p>0.05 [16]. The model was developed on a concatenated data set from TRAIN and TEST subsamples. The efficiency of the obtained logistic model was assessed similarly to that of ANN.

### Independent validation

The obtained models were validated using VALID subsample. DeLong test was used to compare AUC of the models.

## Results

Hospital mortality in the sampling under study was 3.8% (213 patients from 5621 died). Table 1 demonstrates the comparison of patients with and without CMVO. CMVO group was found to have a higher hospital mortality rate and more patients with such CMVO predictors as age, MI with ST-segment elevation, severe CHF, ineffective thrombolytic therapy, marked IRA thrombosis, more marked atherosclerotic CA, a great number of stents, and single-step stenting of several CA. However, symptom-to-balloon time in this group was less.

Table 2 represents training outcomes, ANN precision and validation assessment, as well as the development and validation of a logistic regression model.

**Table 2:** Results of development, training, and validation of models

Metrics	ANN training result (TRAIN)	ANN test result (TEST)	ANN validation result (VALID)	Regression model	
				Initial result	Validation result
				(TRAIN + TEST)	(VALID)
Area under ROC curve, 95% CI	0.69 (0.68–0.71)	0.73 (0.70–0.77)	0.64 (0.61–0.67)	0.71 (0.70–0.73)	0.64 (0.61–0.67)
Cut-off value of model result	>0.4928	>0.4928	>0.4928	>–2.705	>–2.705
True positive cases (n)	49	6	13	57	10
False positive cases (n)	399	72	76	456	74
True negative cases (n)	3518	620	735	4153	737
False negative cases (n)	94	19	20	111	23
Sensitivity (%)	34.3	24.0	39.4	33.9	30.3
Specificity (%)	89.8	89.6	90.6	90.1	90.9
Positive predictive value (%)	10.9	7.7	14.6	11.1	11.9
Negative predictive value (%)	97.4	97.0	97.4	97.4	97.0
*F*_1_-score (%)	16.6	11.7	21.3	16.7	17.1

Here: ANN — artificial neural network.

**Figure 3.**
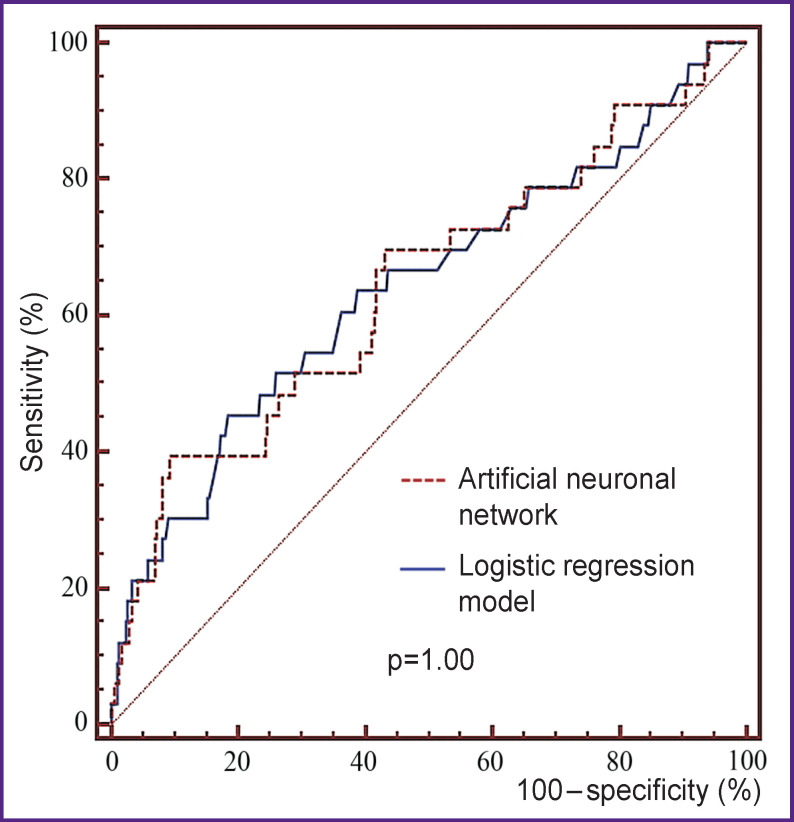
Comparison of ROC curve models on VALID subsample

Comparison of AUC obtained using ANN and logistic regression on VALID subsample (Figure 3) showed no statistically significant differences (p=1.00).

## Discussion

Due to the use of a retrospective analysis and associated limitation of the available information, CMVO was stated only according to TIMI flow grade in IRA after PCI. Thus, most severe CMVO cases were studied, their frequency in practice was relatively few (4% in the present study). Severe CMVO was associated with “classical” predictors and increased hospital mortality risk. Less symptom-to-balloon time in CMVO patients was likely due to patients with non-ST-segment elevation MI included in the study. Such patients came to surgery later and have lower CMVO risk.

CMVO syndrome pathogenesis is known to be highly heterogeneous. The phenomenon can result from distal thrombotic microembolization, ischemic perfusion impairment, and endothelial dysfunction a patient initially had against the background of accompanying pathology or the presence of some variants of single nucleotide gene polymorphism [2]. Due to this, every patient can exhibit a various combination and intensity of the above-described pathogenic mechanisms; that, in its turn, causes some difficulties in CMVO and underlines the need for independent data validation [17].

The attempts to unite within one model the characteristics presenting all pathogenic CMVO elements result in model overcomplication and difficulties of its effective usage in real clinical practice. So, for a famous model by Wang [3], the laboratory findings are needed, which are rarely available during an operation on patients with MI due to the necessity to perform emergency PCI immediately.

Within the terms of the present study, we attempted to develop a prognostic model combining simplicity (the use of indices, which are routinely available when choosing a surgical approach), adequate accuracy (a result should have some clinical value), and proved efficiency (the presence of validation on independent data). Such requirements, to a large extent, determined the choice of ANN to develop a prognostic model.

ANN is known to enable to find complex dependencies and classify based on a variety of features, which are poorly distinguished using other analyses [8, 11]. This ANN property was considered most important under the terms of unavailability when predicting a number of highly specific CMVO characteristics. However, when performing a comparative analysis of the findings, the accuracy of a logistic regression model was found to be in many respects compatible with the accuracy of a developed ANN. So, AUC obtained in VALID subsample was the same (see Figure 3). Apparently, the fact can be explained by insufficient specificity of available characteristics in relation to CMVO. The lack of input data of accurate laboratory markers made the objective difficult and prevented ANN from realizing its potential in classifying complex regularities. Moreover, low frequency of CMVO in the sampling under study led to the problem of imbalanced data [8, 10]; the problem consisted in ignoring ANN of minority class (patients with CMVO) when training. The problem was solved using the techniques for imbalance correction and using special metrics (*F*_1_-score).

Most likely, the above-mentioned difficulties of CMVO pathogenesis, imbalance, and low specificity of input data are also the main reasons of moderate accuracy of the developing models in general (see Table 2). It is important to emphasize that despite all restrictions, an obtained ANN is not devoid of clinical value, and has a number of advantages over a regression model. Considering high negative predictive value (97.4%) excluding CMVO, a predicted ANN result gives a surgeon the confidence in safety when performing highly technical and “aggressive” PCI, if it is necessary (the use of several stents, post-dilatation with high-pressure balloon, etc.) [18]. On the contrary, quite modest positive predictive value (14.6%) taking into account low CMVO frequency (4%) makes the fact of obtaining the data beneficial enough. In this case, a doctor has extra reasons to perform a minimally invasive procedure, as well as to overestimate risks and administer some additional treatment, e.g., IIb/IIIa-platelet glycoprotein receptor blockers [19]. It is essential that validation showed ANN to have higher *F*_1_-score values — 21.3% versus 17.1% in a regression model suggesting its higher accuracy under conditions of imbalanced data. It should be noted that an advantage of the obtained models is validation on independent data, and it increases their reliability. A developed ANN can be considered a useful additional instrument when making a decision on an optimal surgical approach in MI patients.

### Study limitations

The present study and a developed model have some limitations. Retrospective nature of characteristics and the limitations of the used database prevented from including a number of potential CMVO predictors into a model (e.g., collateral blood flow intensity), as well as using wide-ranging criteria for diagnostic outcomes (myocardial perfusion by myocardial blush grade (MBG) and ST-segment on an electrocardiogram). According to initial inclusion criteria, a model can be used only for patients admitted with MI diagnosis. Moderate accuracy characteristics of the model require its careful usage. It is reasonable to carry out an extra prospective validation using samplings from other medical institutions.

## Conclusion

A developed artificial neural network uses routinely available characteristics when choosing a surgical approach and enables to predict the development of coronary microvascular obstruction (no-reflow) when performing percutaneous coronary interventions in patients with myocardial infarctions with accuracy sufficient for practical application (the area below ROC curve is 0.69). An essential advantage of a developed model is its validation on independent data (the area below ROC curve is 0.64; negative predictive value is 97.4%; positive predictive value is 14.6%).
